# Effects of 1-stage revascularization and temporary external fixation combined with 2-stage Ilizarov technique in the treatment of bone defects in lower limb destruction injury: A case report

**DOI:** 10.1097/MD.0000000000030149

**Published:** 2022-08-19

**Authors:** Ying-Jie Xu, Xu Gao, Hao Ding, Xian-Min Bu, Hai-Bin Wang, Bin Wu

**Affiliations:** a Department of Clinical Medicine, Jining Medical University, Jining City; b Department of Qingdao Medical College, Qingdao University, Qingdao City; c Department of Pathology, Jining No. 1 People’s Hospital; d Department of Orthopedics, Affiliated Hospital of Jining Medical University, Jining City, China.

**Keywords:** external fixation, Ilizarov bone transport, limb bone defect, reascularization

## Abstract

**Rationale:**

To evaluate the clinical effects of 1-stage revascularization, vacuum sealing drainage covering the wound, temporary external fixation and 2-stage Ilizarov bone transport for the treatment limb destruction injury.

**Patient concerns and diagnosis:**

Nine patients with limb destruction injury between September 2014 and June 2019 at our institute were evaluated retrospectively. The age of patient was 21 to 51 years with an average of 33 years. The injuries were caused by vehicle accidents in 4 patients, gunshot in 1 patient, and crushing injuries in 4 patients. All of them had vascular injury. The average length of bone defect was 9.5 (8.3–10) cm. Regular follow-up was performed on wound healing, bone transport time, bone healing time, external fixation index, and limb function.

**Interventions:**

All patients underwent 1-stage revascularization and temporary external fixation during emergency surgery, and then gradual bone transport by Ilizarov fixator was performed until the broken fracture site was reunited.

**Outcomes:**

Nine patients were followed up for 12 to 48 months (average 30 months). Six patients were treated with autogenous cancellous bone graft for the second time, and 2 patients healed spontaneously. The mean wound healing time was 86 (73–90) days. The bone transport time was 97 (88.3–105.3) days, and the bone mineralization time was 164.5 (156.8–181.3) days, and the healing time of the docking sites was 6.8 (6.1–8.3) months. The external fixator time was 14.5 (12.5–17) months with the external fixation index was 1.5 (1.4–1.8) m/cm. At the last follow-up, according to the Association for the Study of the Method of Ilizarov functional scores, excellent functional outcomes were obtained in 5 patients, good in 1 patients, moderate in 2 patients. According to the Association for the Study of the Method of Ilizarov Radiological System, excellent functional outcomes were obtained in 6 cases and good in 2 cases.

**Lessons:**

One-stage revascularization and temporary external fixation combined with 2-stage Ilizarov bone transport technique for the treatment of bone defects in limb destruction injury have satisfactory clinical effects and few complications, and can be applied under the condition of strict understanding of surgical indications.

## 1. Introduction

As a special type of high-energy injury, limb destruction injury is more serious than Gustilo IIIc open fracture injury.^[1]^ It refers to the extensive damage of major blood vessels, nerves, bones and soft tissues of limbs, and cannot be repaired or retransplanted normally.^[2]^ Although several young patients with complete distal limb structure, limb salvage or amputation in the treatment of limb destruction injury is still controversial.^[3]^ Therefore, the present study aimed to retrospectively analyze the clinical effects of 9 patients with limb destruction injury treated by 1-stage revascularization and external fixation combined with 2-stage Ilizarov technique for bone transport.

## 2. Patients and Methods

### 2.1. Inclusion and exclusion criteria

Inclusion criteria are as follows: patients had limb destruction injury with intact distal leg and ankle; tibial nerve integrity; and cases with complete follow-up.

Exclusion criteria are as follows: patients with severe brain, chest, and abdomen injuries; limb ischemia time was more than 8 hours; and associated with serious diabetes, hypertension, heart disease, or other diseases.

### 2.2. The general information

According to the inclusion and exclusion criteria, a total of 9 patients with lower limb destruction injury were included and analyzed in our hospital from September 2014 to June 2019. Patients were aged 21 to 51 years with an average age of 33 years. All patients had severe open injuries of unilateral limbs with large bone defects and main artery vessels injury. Emergent treatments including bandaging, antibiotics, and tetanus prevention were given to all patients. The time from injury to operation was 3 to 8 hours, with an average of 6.3 hours. This study was approved by the medical ethics committee of our institution and informed consent was obtained from all patients.

### 2.3. Surgical method

**Figure 1.**
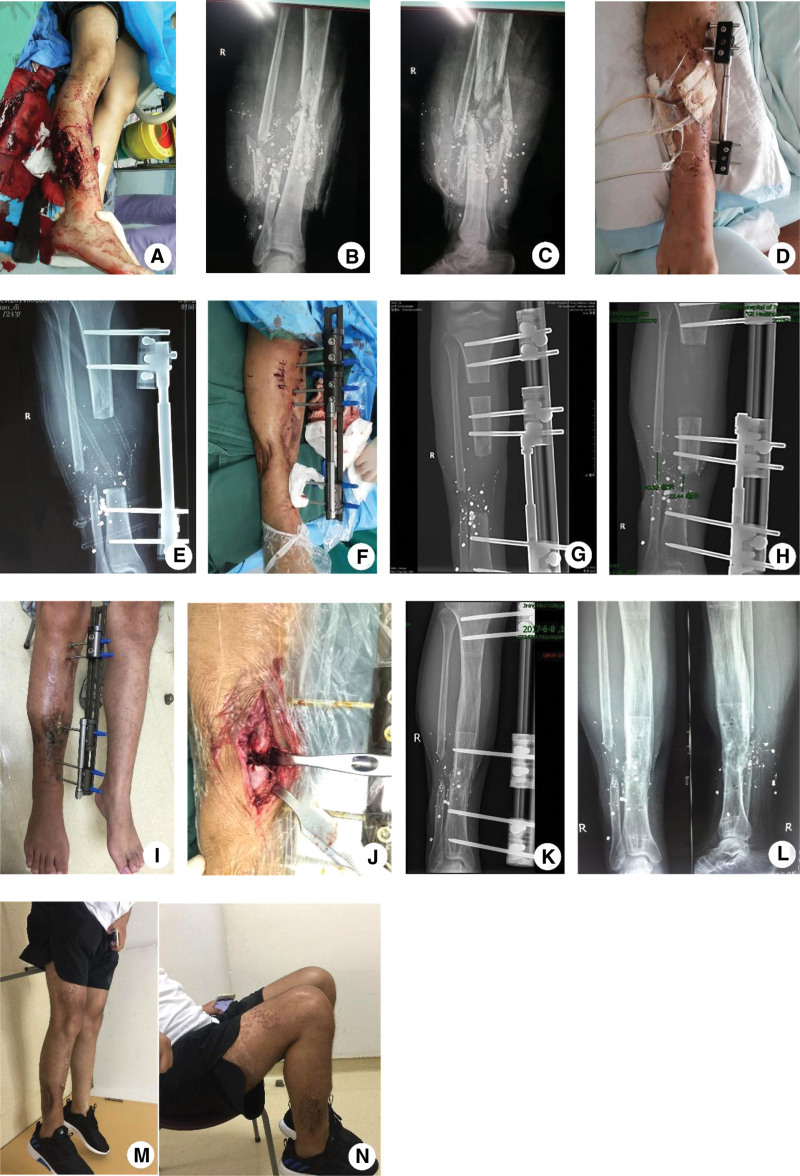
(A, B, C) A 24-yr-old man sustained open injuries in the right lower limb caused by gunshot. (D, E) Debridement, temporary fixation of external fixator, and VSD of the wounds were carried out. (F, G, H) A Ilizarov unilateral external fixator was used and bone transport were performed, and the length of the tibial defects was approximately 10 cm. (I, J) The docking sites did not heal after bone transport. The docking sites were cleared and cancellous bone grafted. (K) At 4 mo after bone graft, X-ray examination showed healing of the bone fracture, and the external fixator was scheduled to be removed. The postoperative external fixation time was 20 mo, and the external fixation index was 2.0. (L, M, N) Clinical appearance and X-ray findings 4 mo after external fixator was removed. The postoperative ASAMI functional score of the affected limb was excellent. ASAMI = Association for the Study of the Method of Ilizarov, VSD = vacuum sealing drainage.

The patients were taken to the operating room and subjected to general anesthesia. Subsequently, debridement and irrigation on the wound to remove contaminated and severely damaged skin and inactivated soft tissues. In all patients, the exposed bone defect length was approximately 9.5 (8.3–10) cm after removal of the fracture fragments. First, temporary external fixator was used to fix the fracture site and the muscle tissue around the blood vessel was preserved as much as possible. The artery on the injured side was repaired with the great saphenous vein of the opposite limb. The remaining vein of the injured limb was also ligated. Next, part of the incision was sutured under tension-free conditions, while the remaining incision that could not be closed was covered with vacuum sealing drainage. Skin grafting was given to the skin defect area. The Ilizarov technique was applied after surgery 3 to 4 weeks. We removed the original external fixator, and then set 3 fixation points for the Ilizarov fixator, namely the proximal fixation module, the intermediate bone transport module, and the distal tibial fixation module. Between the proximal fixation module and intermediate bone transport module, the tibia was cut using wire saw (Fig. 1G). Following that X-ray fluoroscopy confirmed the force line of the tibia and the position of external fixation was good, the operation was finished. A week later, gradual tibial transport by Ilizarov external fixator was performed until the broken fracture site was reunited. During the postoperative observation period, if there is no sign of bone healing at the fracture end, bone graft was performed.

### 2.5. The evaluation index

Average operation times, wound healing time, bone removal time, recontact healing time, mineralization time of regeneration area, wearing time of external fixator, and external fixation index were evaluated. Association for the Study of the Method of Ilizarov (ASAMI) score was used to assess functional outcomes.^[4,5]^

## 3. Results

In our study, 9 patients were followed up for 12 to 48 months with an average of 30 months. Two cases healed spontaneously, and 6 cases underwent bone graft. During the follow-up, 1 elderly patient refused to limb salvage and underwent amputation 2 weeks after injury. The remaining 8 patients underwent operations about an average of 6. Six patients were treated skin graft and 2 cases underwent flap transplantation. The mean wound healing time was 86 (73–90) days. The bone transport time was 97 (88.3–105.3) days with the bone mineralization time was 164.5 (156.8–181.3) days. The healing time of the docking sites was 6.8 (6.1–8.3) months. The external fixator time was 14.5 (12.5–17) months, and the external fixation index was 1.5 (1.4–1.8) m/cm. Pin tract infections occurred in 7 patients and resolved after oral antibiotics and care of the pin sites. Delayed healing occurred in 6 patients and resolved after autogenous cancellous bone grafting. Four patients had different degree of foot drop accompanied with limping. At the last follow-up, according to the ASAMI functional scoring system, excellent functional outcomes were obtained in 5 patients, good in 1 patient and moderate in 2 patients. According to the ASAMI radiological system, they were excellent in 6 cases and good in 2 cases. A representative case is shown in Figure 1.

## 4. Discussion

### 4.1. Choice of treatment for limb destruction injury: Limb amputation or salvage

Limb destruction injury is a type of severe limb trauma involving the skin, blood vessels, nerves, and bones. Whether the patient should be amputated or salvaged at an early stage is still controversial.^[6]^ At present, various assessment systems have been reported for assessing limb salvage or amputation, but there is no single system can be as the best judgement for a particular case. Therefore, clinical treatment is still mainly based on the location of the limb injury, technical level, patient’s economic conditions, and requirements.^[7]^ In our study, a 51-year-old elderly patient strongly requested limb salvage after injury, and then recovered well after treatment. However, given that the long time for bone transfer and the high cost of treating, we eventually performed amputation for that patient after 2 weeks. For the remaining 8 young patients with single injuries, the ischemia time was <8 hours and the bottom of the plantar sensation remained good. Therefore, patients with limb destruction injury should be fully evaluated and communicated before surgery, and individualized treatment plans should be formulated according to the degree of injury such as fractures, blood vessels, nerves, and tendons.

### 4.2. Advantages and disadvantages of temporary external fixator

The soft tissue of the injured limb is usually damaged seriously. Temporary external fixation was able to stabilize the fracture site, and avoid more damage to the surrounding soft tissues to enhance the survival rate of the injured soft tissues.^[8]^ However, it still remains debates on whether temporary external fixation was more applicable than Ilizarov technique for patients. Temporary external fixation was more briefness, shorter operation time, and lower cost. However, external fixation eventually needs to be replaced by Ilizarov technique in the 2-stage treatment. In recent years, more evidences have reported that patients with open fractures and bone defects were treated with 1-staged Ilizarov external fixation.^[9,10]^ For emergency patients, although Ilizarov technique is more stable than temporary external fixator, the installation time of fixator was too long and it needs repeated fluoroscopy to adjust the limb force line. Therefore, Ilizarov technique seems not suitable for emergency surgery for emergency patients with severe injury. In addition, the cost of Ilizarov external fixator is higher. Once the limb salvage was failure, it required amputation and caused dissatisfaction among patients. In our study, we selected temporary external fixation in early treatment stage, and then changed to the final Ilizarov external fixator after limb salvage was succeed.

### 4.3. Advantages and key points of Ilizarov technique in repairing bone defects

In the early 20th century, based on the tension-stress principle, Ilizarov technique was widely applied in the treatment of bone defects, which greatly improved the rate of limb salvage.^[11]^ In the present study, following Ilizarov external fixator treatment, 8 patients with bone defects obtained good results and only remained fewer limb dysfunction. We consider that Ilizarov technique in repairing limb bone defects has the following advantages. First, Ilizarov technique has advantage of minimally invasive, no excessive stripping of soft tissues, and protection of local blood supply. Second, the ring external fixator is stable and can provide axial stress, eliminate torsion and shear stress in all directions of the limb, which stimulates formation of callus and reduce the occurrence of bone nonunion. Finally, Ilizarov technique allowed patient early postoperative rehabilitation exercise and improves the range of motion.^[12–14]^ There are 2 types for Ilizarov fixator including ring fixator and unilateral external fixator. For tibial bone defects, both ring fixator and unilateral external fixator have certain advantages. For femoral bone defects, only unilateral fixator can be applied because of the surrounding muscle hypertrophy. In our study, 1 case of femoral bone defect was fixed with unilateral external fixator and the remaining patients with tibial bone defect were treated with ring fixator and unilateral external fixator, respectively. We found that the ring fixator has more advantages in stability and force line. Although Ilizarov technique has more benefits,^[15]^ there is still remaining several problems to be paid attention in clinical work. For example, Ilizarov technique has a long treatment cycle for bone defects, which is not suitable for the elderly.^[16–18]^

## 5. Conclusions

To sum up, the treatment of limb destruction injury is still full of challenges. We suggest that 1-stage revascularization combined with temporary external fixation has the advantages of rapidness, effectiveness, and low cost. After patient in stable condition, 2-stage Ilizarov technique can be used in the treatment of bone defects in limb destruction injury. However, due to lack of cases compared with other limb salvage procedures, multi-center study and more patients are needed to further evaluate the effects of our study.

## Acknowledgments

The authors thank the department colleagues and the patients for their dedication, and the patients had signed the informed consent forms.

## Author contributions

Conceptualization: Bin Wu.

Data curation: Hai-Bin Wang.

Formal analysis: Ying-Jie Xu, Hao Ding.

Investigation: Bin Wu.

Writing—original draft: Ying-Jie Xu, Hao Ding.

Writing—review & editing: Xu Gao, Xian-Min Bu, Bin Wu.

The authors have no conflicts of interest to disclose.
